# A fluorescent probe for detection of Hg^2+^ ions constructed by tetramethyl cucurbit[6]uril and 1,2-bis(4-pyridyl)ethene

**DOI:** 10.3762/bjoc.19.63

**Published:** 2023-06-13

**Authors:** Xiaoqian Chen, Naqin Yang, Yue Ma, Xinan Yang, Peihua Ma

**Affiliations:** 1 Key Laboratory of Macrocyclic and Supramolecular Chemistry of Guizhou Province, Guizhou University, Guiyang 550025, China; 2 Guiyang College of Humanities and Science, Guiyang 550025, China

**Keywords:** 1,2-bis(4-pyridyl)ethane, fluorescent probe, Hg^2+^ ion recognition, host–guest chemistry, tetramethyl cucurbit[6]uril

## Abstract

In this paper, tetramethyl cucurbit[6]uril (TMeQ[6]) and 1,2-bis(4-pyridyl)ethene (G) were used to construct a supramolecular fluorescent probe G@TMeQ[6]. The host–guest interaction between TMeQ[6] and G was investigated using ^1^H NMR spectroscopy, single-crystal X-ray diffraction and various experimental techniques. The results show that TMeQ[6] and G form an inclusion complex with a host–guest ratio of 1:1 and the equilibrium association constant (*K*_a_) was 2.494 × 10^4^ M^−1^. The G@TMeQ[6] fluorescent probe can sensitively recognize Hg^2+^ ions by fluorescence enhancement. The linear range is 0.33 × 10^−5^–1.65 × 10^−5^ mol·L^−1^, *R*^2^ = 0.9926, and the limit of detection is 4.12 × 10^−8^ mol·L^−1^. The fluorescent probe can be used to detect the concentration of Hg^2+^ ions in aqueous solution, and provides a theoretical basis for the development of new fluorescent probes for detecting heavy metal ions.

## Introduction

Mercury, as one of the most toxic heavy metal pollutants, not only seriously pollutes the ecological environment but also causes great harm to human health. Mercury and inorganic mercury ions (Hg^2+^) in nature can be converted into organic mercury under the action of microorganisms, which cannot be decomposed or degraded into nontoxic substances in the human body [1–2], which will seriously threaten human health. Therefore, China has formulated water pollutant emission limits [3]. Traditional Hg^2+^ ion detection methods include atomic absorption spectroscopy, mass spectrometry, emission spectroscopy and electrochemical methods [4–7]. These detection methods are expensive and time-consuming, and the detection of samples becomes extremely difficult. Therefore, it is of great significance to construct a fluorescent probe detection system with high sensitivity, a simple detection method and at low costs [8–10].

Cucurbit[*n*]urils (Q[*n*]s) have a highly symmetrical rigid structure and hydrophobic cavities [11–15]. Cavities of different sizes can encapsulate different guest species to form various host–guest inclusion complexes [16–20], which have broad application prospects in supramolecular catalysis [21–23], molecular recognition [24–25], and drug delivery [26–27]. In recent years, in the field of supramolecular chemistry, the detection of analytes based on cucurbit[*n*]uril fluorescent probes has become increasingly mature [9,28–31]. For example, the host–guest fluorescent probe of cucurbit[10]uril (Q[10]) and the fluorescent dye acridine (AD) was used to identify the pesticide dodine (DD) [32]; a fluorescent probe of cucurbit[10]uril (Q[10]) and aminopropyl-1-pyrenebutanamide (PBA) was also constructed to detect Fe^3+^ and Ag^+^ ions in aqueous solution [33], which can be used as a potentially useful fluorescent sensor. Pang and co-workers found that the fluorescent probe of cucurbit[8]uril (Q[8]) and squaraine dye (SQ2) has a high selectivity for Hg^2+^ ions [34]. Because of the synergistic combination of Q[8], SQ2 and Hg^2+^ ions, it shows fluorescence quenching. Cong's group found that Q[7] can encapsulate the benzimidazole part of *N*-(2-benzimidazolylmethyl)-*N*,*N*-bis(2-pyridylmethyl)amine cation (BIBPA^+^) to construct a host–guest fluorescent probe. The change in fluorescence intensity can be used for the recognition of Cd^2+^ and Zn^2+^ ions [35]. The symmetric tetramethyl cucurbit[6]uril (TMeQ[6]) is one of the earliest characterized modified cucurbit[6]urils [36]. Compared with Q[6], tetramethyl cucurbit[6]uril (TMeQ[6]) has good solubility in water, which provides convenience for studying the host–guest chemistry of TMeQ[6] and constructing fluorescent probes in aqueous solution [37–38].

**Figure 1:**
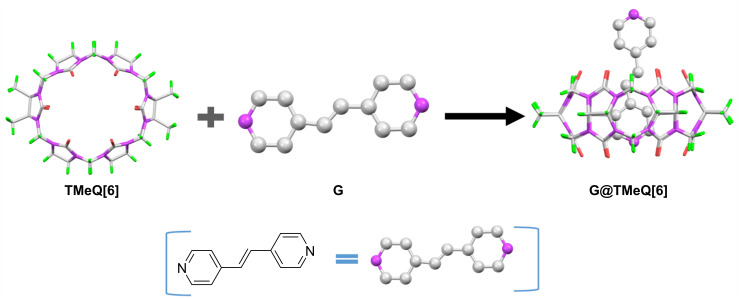
Supramolecular assembly of TMeQ[6] and 1,2-bis(4-pyridyl)ethene (G).

There is a π–π conjugation effect between the carbon–carbon double bond and the pyridine ring in 1,2-bis(4-pyridyl)ethene (G), which determines its ultraviolet absorption [39]. Because the N atom on the pyridine ring of the G molecule has lone-pair electrons, it can form coordination compounds with metal ions. At present, the host–guest fluorescent probes designed by G and Q[*n*]s have been rarely reported. Therefore, we constructed the host–guest fluorescent probes of TMeQ[6] and 1,2-bis(4-pyridyl)ethene (Figure 1). The fluorescence response and mechanism of metal ions were studied. It was found that G@TMeQ[6] had specific recognition of Hg^2+^ ions in an aqueous solution, which provides a theoretical basis for the development of new fluorescent probes for the detection of heavy metal ions.

## Results and Discussion

### The interactions of G with TMeQ[6]

#### UV–vis spectroscopy analysis

**Figure 2:**
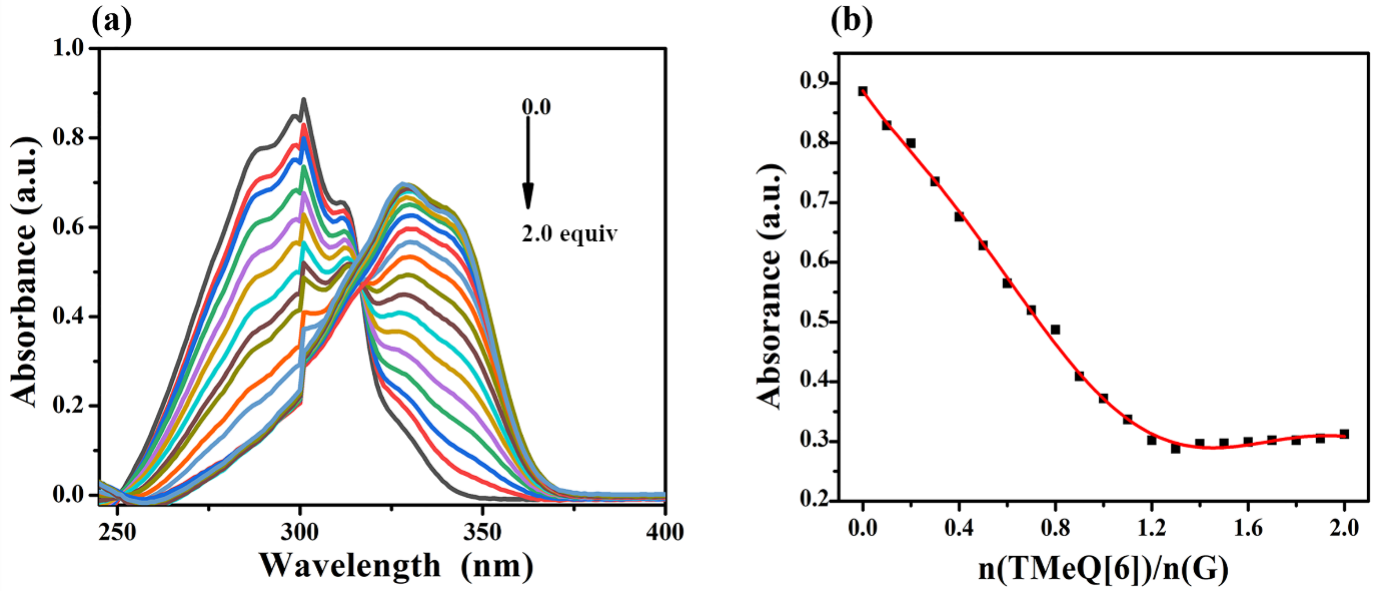
(a) UV–vis titration of G (3.0 × 10^−5^ mol·L^−1^, pH 6.5) in aqueous solution with the increase of TMeQ[6] concentration; (b) plots of *n*(TMeQ[6])/*n*(G) vs ultraviolet absorption of G.

The binding interaction between G and TMeQ[6] in aqueous solution was studied using UV–vis absorption spectroscopy. Figure 2a shows that the absorbance of G decreases with the addition of TMeQ[6], and the wavelength redshifts from 301 nm to 330 nm, indicating that TMeQ[6] has binding affinity for G. The molar ratio method (Figure 2b) shows that when *n*(TMeQ[6])/*n*(G) = 1:1, the absorption value of the system gradually stabilizes, indicating that the guest G and TMeQ[6] form an inclusion complex with a molar ratio of 1:1.

#### Fluorescent spectroscopic analysis

**Figure 3:**
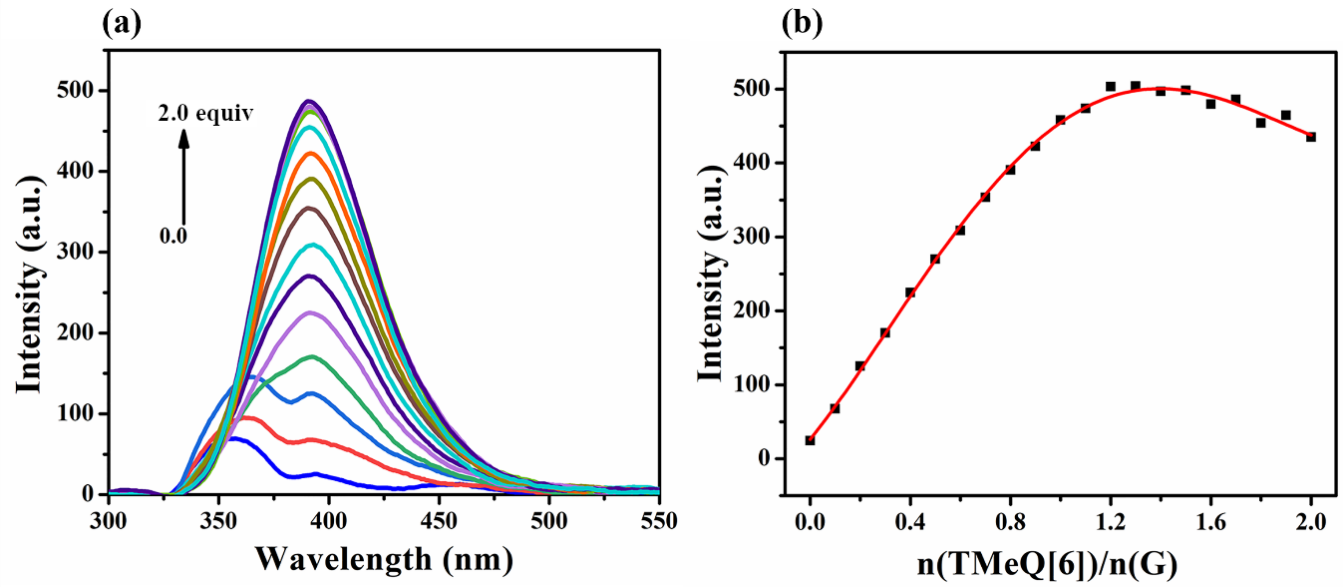
(a) Fluorescence spectra (U = 550 V, pH 6.5) of G (3.0 × 10^−5^ mol·L^−1^) in aqueous solution with increasing TMeQ[6] concentration; (b) plots of *n*(TMeQ[6])/*n*(G) vs fluorescence intensity of G.

The interaction between G and TMeQ[6] in aqueous solution was investigated using fluorescence titration experiments. The fluorescence titration curve (Figure 3a) shows that at the excitation wavelength of 351 nm, G has an emission peak at a wavelength of 350 nm. With the continuous addition of TMeQ[6], the fluorescence intensity of G is continuously enhanced, and the wavelength is redshifted to 391 nm, indicating that TMeQ[6] interacts with the guest molecule G. The TMeQ[6] cavity may limit the rotation of the pyridine ring on the G molecule, and form an effective conjugated system with another pyridine ring outside the cavity, resulting in enhanced fluorescence [40]. The molar ratio curve (Figure 3b) shows that when TMeQ[6] is added up to 1.0 equiv, the fluorescence intensity of the system gradually stabilizes, confirming that a G and TMeQ[6] inclusion complex is formed with a 1:1 stoichiometry.

#### Isothermal titration calorimetry (ITC) analysis

The association constant and thermodynamic parameters of the host–guest interaction between G and TMeQ[6] can be obtained using ITC. At 25 °C, a neutral aqueous solution of TMeQ[6] (1.0 × 10^−4^ mol·L^−1^, 1.00 mL) was gradually added to the aqueous solution of G, and the exothermic isotherms (Figure S1 in Supporting Information File 1) and thermodynamic data (Table 1) were obtained. The equilibrium association constant (*K*_a_) of G and TMeQ[6] is 2.494 × 10^4^ M^−1^, Δ*H* = −88.43 kJ/mol, which is an exothermic reaction (enthalpy-driven). The results show that the binding ability of G and TMeQ[6] is strong, and the ratio is 1:1. The results are consistent with those obtained by UV–vis spectroscopy and fluorescence spectroscopy.

**Table 1:** Thermodynamic parameters of G/TMeQ[6].

Complex	*K*_a_ (M^−1^)	*n*	∆*H* (kJ·mol^−1^)	*T*∆*S* (kJ·mol^−1^)

G@TMeQ[6]	2.494 × 10^4^	1.040	−88.43	−63.33

#### Single-crystal X-ray diffraction analysis

**Figure 4:**
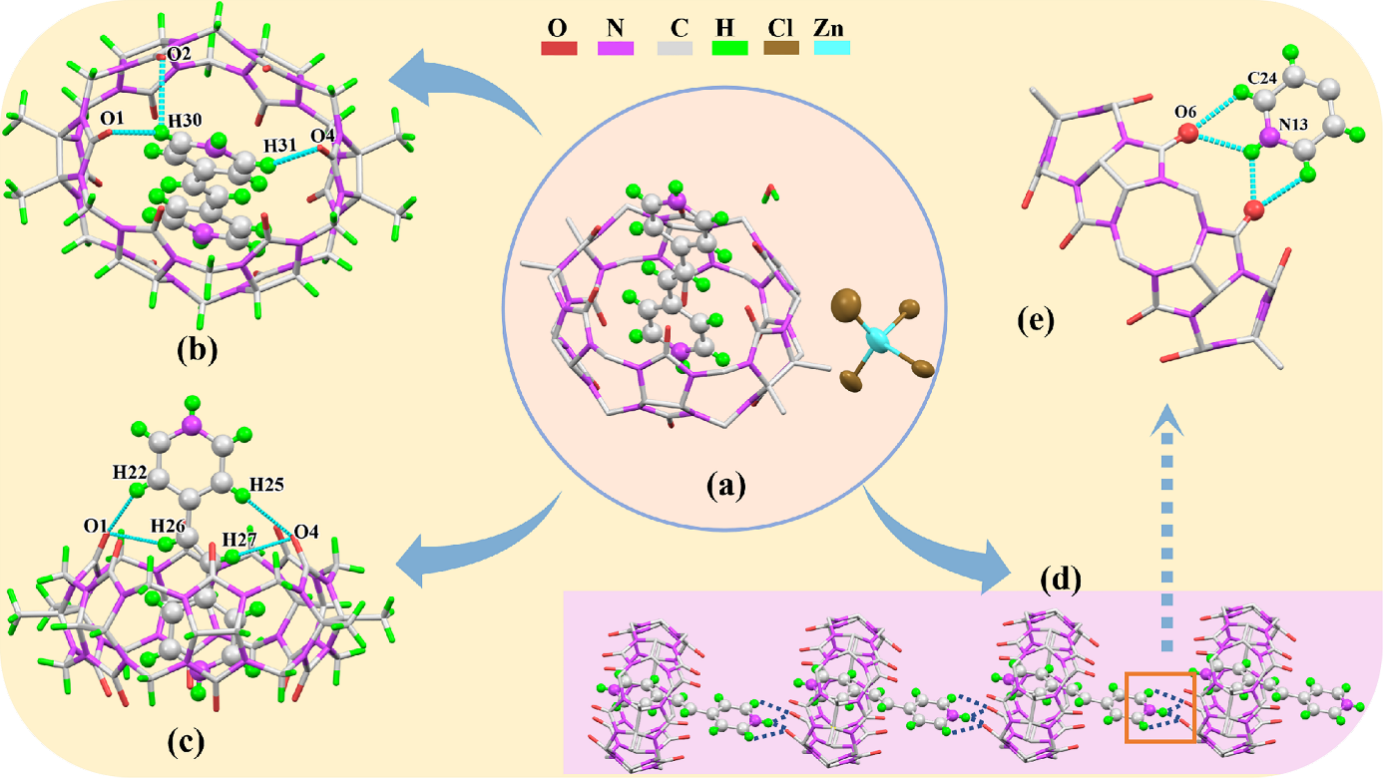
(a) Crystal structure of complex **1**; (b) and (c) the binding mode of G with TMeQ[6]; (d) the supramolecular one-dimensional structure of G@TMeQ[6]; (e) Detailed interactions between adjacent complexes.

The crystal structure of the inclusion complex formed by TMeQ[6] and G was obtained using X-ray single-crystal diffraction analysis. The crystal data and parameters are shown in Table 2. The single-crystal structure determination shows that the inclusion complex crystallizes in the triclinic crystal system, with the chiral space group *P*-1. Figure 4a shows that the basic crystal structure of complex **1** contains a TMeQ[6] molecule, a G molecule, a free water molecule and a [ZnCl_4_]^2−^ anion. It can be clearly seen that one pyridyl group of the G molecule enters the cavity of TMeQ[6], whereas the other pyridyl group is outside the cavity, forming a 1:1 inclusion complex with TMeQ[6]. Figure 4b shows that the hydrogen atoms on the G molecule form C–H30···O1, C–H30···O2 and C–H31···O4 hydrogen bonds with the carbonyl oxygen and carbon atoms on TMeQ[6], and the bond distances are 2.163, 2.707 and 2.228 Å, respectively. In Figure 4c, the hydrogen atoms of G and the carbonyl oxygen of TMeQ[6] form C–H22···O1, C–H26···O1, C–H25···O4 and C–H27···O4 hydrogen bonds with bond distances of 2.370, 2.474, 2.564 and 2.685 Å, respectively. These interactions contribute to the formation of stable inclusion complexes. Figure 4d is a one-dimensional supramolecular chain of G@TMeQ[6], which is composed of hydrogen bonds C24–H···O6 and N13–H···O6 formed by the protons on the pyridyl group outside the cavity and the carbonyl oxygen of the adjacent TMeQ[6] port. The G molecule acts as a medium for connecting two adjacent TMeQ[6].

### The possible mechanism of G@TMeQ[6] to detect Hg^2+^ ions

#### Fluorescence spectroscopy to investigate the specific recognition of Hg^2+^ ions by G@TMeQ[6]

**Figure 5:**
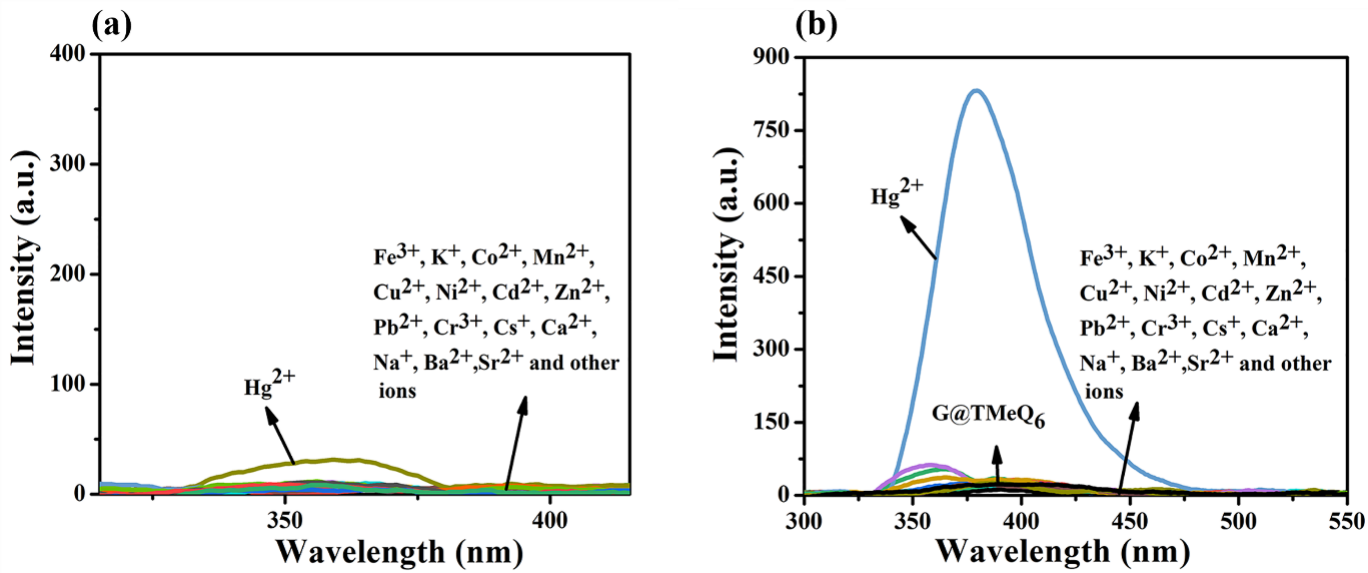
(a) Fluorescence response of G (3.0 × 10^−5^ mol·L^−1^) to metal cations in aqueous solution; (b) fluorescence response (λ_max,em_ = 380 nm) of G@TMeQ[6] (1:1, 3.0 × 10^−5^ mol·L^−1^) to metal cations in aqueous solution. (*c*_(other metal cations)_ = 3.0 × 10^−4^ mol·L^−1^, U = 520 V, pH 6.5).

The fluorescence response of G and G@TMeQ[6] to various metal ions (Fe^3+^, K^+^, Co^2+^, Mn^2+^, Cu^2+^, Ni^2+^, Cd^2+^, Zn^2+^, Pb^2+^, Cr^3+^, Cs^+^, Ca^2+^, Na^+^, Ba^2+^, Sr^2+^, Hg^2+^ and other metal cations) in aqueous solution was investigated using fluorescence spectroscopy. It was found that the G molecule had no specific fluorescence response to the above metal cations (Figure 5a). Interestingly, the addition of the Hg^2+^ ion to the G@TMeQ[6] system caused a strong fluorescence enhancement at an excitation wavelength of 351 nm (Figure 5b). The fluorescence intensity at the emission wavelength of 380 nm was enhanced from 25.8 to 831.2, indicating that the G@TMeQ[6] probe has a specific recognition of Hg^2+^.

#### Fluorescence spectroscopy to investigate the anti-interference and competitive ability of the probe to detect Hg^2+^ ions

**Figure 6:**
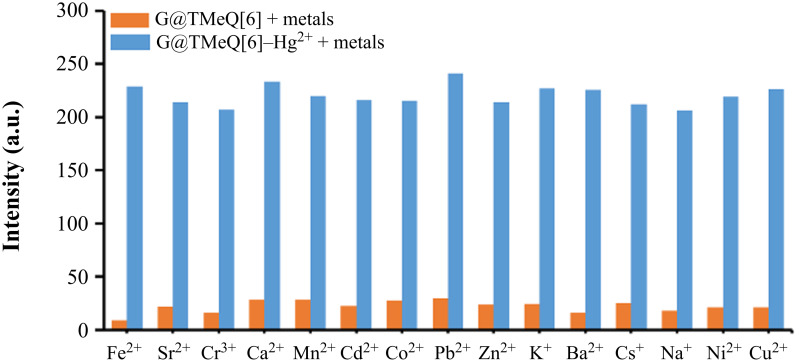
Influence of coexisting ions on Hg^2+^ detection by G@TMeQ[6].

The metal cation interference experiment was used to investigate whether the G@TMeQ[6] fluorescent probe can selectively detect Hg^2+^ ion in the presence of other metal cations (Figure 6). The fluorescence intensity of the system was determined by adding other metal cations to the G@TMeQ[6]–Hg^2+^ system. The results showed that in the presence of other metal ions, the G@TMeQ[6] probe still showed specific recognition of Hg^2+^ ions.

#### Fluorescence spectroscopy analysis of the interaction between the probe and Hg^2+^ ions

**Figure 7:**
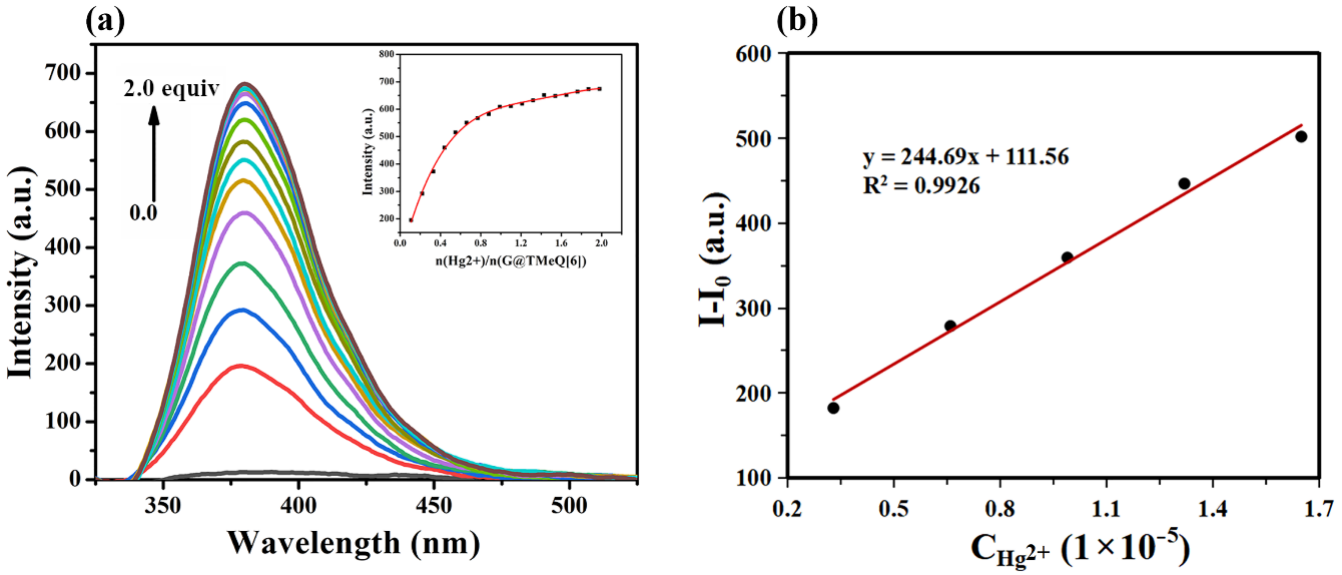
(a) Fluorescence titration curve (U = 500 V, pH 6.5) of probe G@TMeQ[6] (3.0 × 10^−5^ mol·L^−1^) to Hg^2+^ ions; (b) detection limit of probe G@TMeQ[6] to Hg^2+^ ions.

The fluorescence titration curve of G@TMeQ[6] and Hg^2+^ ions showed that the fluorescence intensity of the probe at 380 nm increased with the increase of Hg^2+^ ion concentration (Figure 7a). When *n*(TMeQ[6])/*n*(G) = 1:1, increasing the concentration of Hg^2+^ ion will not lead to a significant change in fluorescence intensity, indicating that the guest molecule G and TMeQ[6] form an inclusion complex with a molar ratio of 1:1. As shown in Figure 7b, when the concentration range of Hg^2+^ ions is 0.33 × 10^–5^–1.65 × 10^–5^ mol·L^−1^, the fluorescence enhancement of the system has a good linear relationship with the concentration of Hg^2+^ ion. The linear regression equation is *y* = 244.69*x* + 111.56, *R*^2^ = 0.9926, and the detection limit (LOD = 3σ/*K,* here σ is the standard deviation and *K* is the slope of the calibration curve) is 4.12 × 10^–8^ mol·L^−1^. The G@TMeQ[6] fluorescent probe can effectively detect Hg^2+^ ions in an aqueous solution.

#### ^1^H NMR spectroscopic titration analysis of the interaction between the probe and Hg^2+^ ions

**Figure 8:**
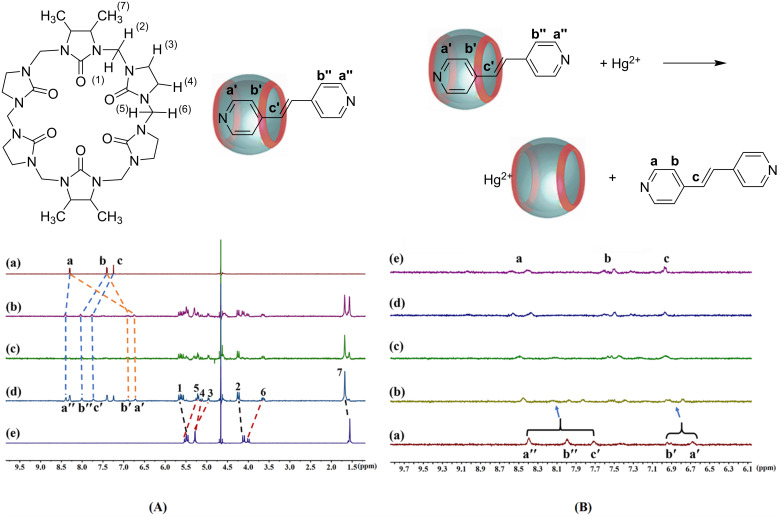
(A) Titration ^1^H NMR spectra (400 MHz) obtained for TMeQ[6] with (a) 0.00, (b) 0.50, (c) 1.00 and (d) 1.20 equiv of G and (e) neat TMeQ[6] (5.0 × 10^−4^ mol·L^−1^); (B) titration ^1^H NMR spectra obtained for G@TMeQ[6] (5.0 × 10^−4^ mol·L^−1^) with (a) 0.00, (b) 0.40, (c) 0.80, (d) 1.00 and (e) 1.4 equiv of Hg^2+^ at 20 °C.

To study the solution complexation between G and TMeQ[6], the ^1^H NMR titration spectrum of TMeQ[6] with different equivalents of guest molecule G was obtained (Figure 8A). When 1.0 equiv G was added, it was found that the proton peak of the G molecule was split, and the chemical shift values of protons Ha′ and Hb′ were shifted upfield by 1.58 and 0.51 ppm, respectively, indicating that some protons on the G molecule entered the TMeQ[6] cavity and were shielded by the cavity. The chemical shift values of the protons Ha″, Hb″ and Hc′ shift downfield by 0.11, 0.62 and 0.50 ppm, respectively, indicating that the protons of this part are located at the port of the TMeQ[6], which is affected by the carbonyl oxygen of the TMeQ[6] port. When the G molecule is added to it, the free proton peak of the G molecule will be observed, indicating that the G and TMeQ[6] form a 1:1 host–guest complex. The proton peaks on TMeQ[6] also split. The protons H1, H2 and H7 shift downfield by 0.11, 0.13 and 0.11 ppm, respectively, whereas the protons H3, H4, H5 and H6 shift upfield by 0.33, 0.15, 0.27 and 0.34 ppm, respectively. The above shows that the guest molecule partially enters the cavity of TMeQ[6].

The interaction mechanism between the fluorescent probe G@TMeQ[6] and Hg^2+^ ion was studied using ^1^H NMR titration experiments (Figure 8B). After the addition of Hg^2+^ ions to the G@TMeQ[6] system, the proton peaks Ha′, Hb′ and Hc′ on the guest molecules in the TMeQ[6] cavity move downfield until they disappear and become free guest proton peaks Ha, Hb and Hc, respectively. The protons Ha″ and Hb″ that did not enter the cavity also gradually moved downfield with the continuous addition of Hg^2+^ ions. When 1.0 equiv. of Hg^2+^ ions were added, they also became a free G molecule with proton peaks Ha, Hb. This indicates that Hg^2+^ ions may coordinate with the port of TMeQ[6] to form a 1:1 coordination compound, which has a competitive effect with the G molecule. The G molecule wrapped in the cavity of the melon ring is squeezed out, and the proton peak of the G molecule will split, indicating that the TMeQ[6] also affects it.

## Conclusion

We studied the host–guest interaction between G and TMeQ[6] and the specific recognition of Hg^2+^ ions by the G@TMeQ[6] fluorescent probe. The interaction ratio of G to TMeQ[6] was 1:1 and the association constant was 2.494 × 10^4^ M^−1^. The interaction ratio between the G@TMeQ[6] probe and Hg^2+^ was proved to be 1:1 using the molar ratio method and ^1^H NMR titration spectroscopy. The recognition mechanism may be that there is a competitive effect between the Hg^2+^ ion and the G molecule, which squeezes out part of G in the TMeQ[6] cavity and coordinates with the port of TMeQ[6]. The detection limit of probe G@TMeQ[6] for Hg^2+^ ions is 4.12 × 10^−8^ mol·L^−1^. The fluorescent probe can effectively identify Hg^2+^ ions and can be used for the detection of Hg^2+^ ions in water.

## Experimental

### Materials

1,2-Bis(4-pyridyl)ethene (G) was purchased from Aladdin, and the other reagents were analytical grade and ready for use. TMeQ[6] was synthesized and purified in our laboratory.

### Preparation of complex **1**

TMeQ[6] (15 mg, 14.26 µmol), ZnCl_2_ (5 mg, 36.7 μmol) and 1,2-bis(4-pyridyl)ethene (3 mg, 16.48 µmol), were dissolved in 5 mL of 3 mol·L^−1^ hydrochloric acid solution, heated at 70 °C, cooled and filtered. The filtrate was slowly evaporated in air (about 10 days) to obtain colorless crystals.

### Ultraviolet–visible absorption and fluorescence spectroscopy

An aqueous solution of TMeQ[6] and G@TMeQ[6] at 3.0 × 10^−5^ mol L^−1^ was prepared. The host–guest interaction between TMeQ[6] and G was investigated using a UV-2700 dual-beam ultraviolet–visible (UV–vis) spectrophotometer and Varian Cary Eclipse fluorescence spectrophotometer at room temperature [41]. At U = 550 V, slit = 5/5, different concentrations of G were added to the TMeQ[6] solution to determine the fluorescence intensity. At the same time, the interaction between the G@TMeQ[6] fluorescent probe and Hg^2+^ ions was studied using fluorescence spectroscopy [29]. The fluorescence intensity of the system was determined by adding different metal ions (3.0 × 10^−4^ mol·L^−1^) to the G@TMeQ[6] aqueous solution at U = 520 V, slit = 5/5 and adding different equivalents of Hg^2+^ to G@TMeQ[6] aqueous solution at U = 500 V, slit = 5/5.

### Isothermal titration calorimetry (ITC)

The neutral aqueous solution of TMeQ[6] (1.0 × 10^–4^ mol·L^−1^, 1.00 mL) was placed in a sample tank, and G solution (1.0 × 10^–3^ mol·L^−1^) was taken in a 250 μL syringe. The temperature was set at 25 °C, titrated 30 times (8 μL each time), and the titration time interval was 300 s. The thermodynamic parameters of each system were measured on a Nano ITC Isothermal Titration Calorimeter. After deleting the first unwanted data point, the data was analyzed using the independent model Launch Nano analysis software.

### ^1^H NMR spectroscopy

The host–guest interaction between G and TMeQ[6] and the interaction mode between G@TMeQ[6] fluorescent probe and Hg^2+^ ion were studied by ^1^H NMR titration spectroscopy. All ^1^H NMR spectroscopy data were recorded on a JEOL JNM-ECZ400s spectrometer in D_2_O at 293.15 K [42].

### X-ray crystallography

Using single-crystal X-ray diffraction has been previously described in the literature [43]. The main crystal structure parameters are recorded in Table 2. In addition, CCDC-2225763 contains the supplementary crystallographic data for this paper. These data can be obtained free of charge from The Cambridge Crystallographic Data Centre via https://www.ccdc.cam.ac.uk/data_request/cif.

**Table 2:** Crystal data and structure refinement for G@TMeQ[6].

G@TMeQ[6]			

Empirical formula	C_52_H_58_Cl_8_N_26_O_13_Zn_2_	*D*_c _*(*g∙cm^−3^)	1.505
formula weight	1676.79	*T* [K]	273.15
crystal system	triclinic	μ [mm^−1^]	1.012
space group	*P*-1	unique reflections	6462
*a* (Å)	12.739(4)	observed reflections	17842
*b* (Å)	12.887(4)	parameters	593
*c* (Å)	13.181(4)	*R*_int_	0.0515
α (deg)	84.385(9)	*R* [I > 2σ(I)]^a^	0.1269
β (deg)	78.229(9)	w*R* [I > 2σ(I)]^b^	0.3974
γ (deg)	60.825(7)	*R* (all data)	0.1661
*V* [Å^3^]	1849.6(9)	w*R* (all data)	0.4519
*Z*	1	GOF on *F*^2^	1.160

^a^Conventional *R* on F*hkl*: ∑||*F_o_*| - |*F_c_*||/∑|*F_o_*|. ^b^Weighted *R* on |F*hkl*|^2^: ∑[*w*(*F*_o_^2^* - F*_c_^2^)^2^]/∑[*w*(*F*_o_^2^)^2^]^1/2^.

## Supporting Information

File 1 ITC exothermic isotherms of the interaction formed between G and TMeQ[6].

File 2 Chemical information file of G@TMeQ[6].

File 3 CheckCIF/PLATON report for the cif file of G@TMeQ[6].
