# Supplementary material for: A fluorescent probe for detection of Hg2+ ions constructed by tetramethyl cucurbit[6]uril and 1,2-bis(4-pyridyl)ethene
Source: Beilstein J Org Chem. 2023 Jun 13;19:864–72. doi: 10.3762/bjoc.19.63 (PMC10280054; doi:10.3762/bjoc.19.63)
Supplement: File 1 — ITC exothermic isotherms of the interaction formed between G and TMeQ[6]. [file Beilstein_J_Org_Chem-19-864-s001.pdf]

BEILSTEIN JOURNAL OF ORGANIC CHEMISTRY

Supporting Information

for

A fluorescent probe for detection of Hg^{2+} ions constructed by tetramethyl cucurbit[6]uril and 1,2-bis(4-pyridyl)ethene

Xiaoqian Chen, Naqin Yang, Yue Ma, Xinan Yang and Peihua Ma

Beilstein J. Org. Chem. **2023,** *19,* 864–872. doi:10.3762/bjoc.19.63

ITC exothermic isotherms of the interaction formed between G and TMeQ[6]

License and Terms: This is a supporting information file under the terms of the Creative Commons Attribution License (https://creativecommons.org/licenses/by/4.0). Please note that the reuse, redistribution and reproduction in particular requires that the author(s) and source are credited and that individual graphics may be subject to special legal provisions.
The license is subject to the *Beilstein Journal of Organic Chemistry* terms and conditions: (https://www.beilstein-journals.org/bjoc/terms)

1. Isothermal titration calorimetry

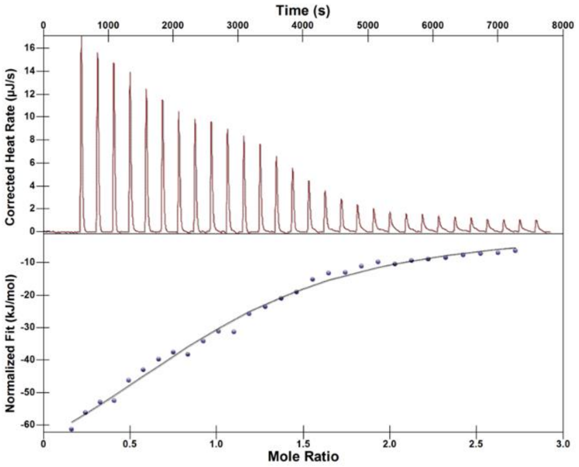

Figure S1: The ITC diagram of the TMeQ[6] to G.
