# Supplementary material for: A fluorescent probe for detection of Hg2+ ions constructed by tetramethyl cucurbit[6]uril and 1,2-bis(4-pyridyl)ethene
Source: Beilstein J Org Chem. 2023 Jun 13;19:864–72. doi: 10.3762/bjoc.19.63 (PMC10280054; doi:10.3762/bjoc.19.63)

BEILSTEIN JOURNAL OF ORGANIC CHEMISTRY

Supporting Information

for

A fluorescent probe for detection of Hg^{2+} ions constructed by tetramethyl cucurbit[6]uril and 1,2-bis(4-pyridyl)ethene

Xiaoqian Chen, Naqin Yang, Yue Ma, Xinan Yang and Peihua Ma

Beilstein J. Org. Chem. **2023,** *19,* 864–872. doi:10.3762/bjoc.19.63

CheckCIF/PLATON report for the cif file of G@TMeQ[6]

License and Terms: This is a supporting information file under the terms of the Creative Commons Attribution License (https://creativecommons.org/licenses/by/4.0). Please note that the reuse, redistribution and reproduction in particular requires that the author(s) and source are credited and that individual graphics may be subject to special legal provisions.
The license is subject to the *Beilstein Journal of Organic Chemistry* terms and conditions: (https://www.beilstein-journals.org/bjoc/terms)

checkCIF/PLATON report

You have not supplied any structure factors. As a result the full set of tests cannot be run.

THIS REPORT IS FOR GUIDANCE ONLY. IF USED AS PART OF A REVIEW PROCEDURE FOR PUBLICATION, IT SHOULD NOT REPLACE THE EXPERTISE OF AN EXPERIENCED CRYSTALLOGRAPHIC REFEREE.

No syntax errors found. CIF dictionary Interpreting this report

Datablock: a_sq

Bond precision:	C-C = 0.0090 A		Wavelength=0.71073
Cell:	a=12.739(4)	b=12.887(4)	c=13.181(4)
	alpha=84.385(9)	beta=78.229(9)	gamma=60.825(7)
Temperature:	290 K		

	Calculated	Reported
Volume	1849.6(10)	1849.6(9)
Space group	P -1	P -1
Hall group	-P 1	-P 1
Moiety formula	C40 H44 N24 O12, C12 H12 N2, 2(Cl4 Zn), 1.4(H2 O) [+ solvent]	2(Cl4 Zn), C40 H44 N24 O12, C12 H12 N2, 1.4(H2 O1)
Sum formula	C52 H58.80 Cl8 N26 O13.40 Zn2 [+ solvent]	C52 H58.80 Cl8 N26 O13.40 Zn2
Mr	1676.42	1676.79
Dx,g cm-3	1.505	1.505
Z	1	1
Mu (mm-1)	1.011	1.012
F000	855.8	856.0
F000’	857.82	
h,k,lmax	15,15,15	15,15,15
Nref	6530	6462
Tmin,Tmax	0.776,0.886	0.585,0.745
Tmin’	0.776	

Correction method= # Reported T Limits: Tmin=0.585 Tmax=0.745
AbsCorr = MULTI-SCAN

Data completeness= 0.990 Theta(max)= 25.026

R(reflections)= 0.1269(4108)		wR2(reflections)= 0.4519(6462)
S = 1.160	Npar= 593	

The following ALERTS were generated. Each ALERT has the format
test-name_ALERT_alert-type_alert-level.
Click on the hyperlinks for more details of the test.

Alert level B

```
PLAT084_ALERT_3_B High wR2 Value (i.e. > 0.25) ...................       0.45 Report
```

Alert level C

```
PLAT077_ALERT_4_C Unitcell Contains Non-integer Number of Atoms ..     Please Check
PLAT082_ALERT_2_C High R1 Value ..................................       0.13 Report
PLAT260_ALERT_2_C Large Average Ueq of Residue Including       Zn1      0.111 Check
PLAT260_ALERT_2_C Large Average Ueq of Residue Including      Zn1A      0.114 Check
PLAT260_ALERT_2_C Large Average Ueq of Residue Including      Zn1B      0.114 Check
PLAT260_ALERT_2_C Large Average Ueq of Residue Including      O01E      0.152 Check
PLAT341_ALERT_3_C Low Bond Precision on  C-C Bonds ...............      0.009 Ang.
PLAT420_ALERT_2_C D-H Bond Without Acceptor  N017     --H01P     .     Please Check
```

Alert level G

```
PLAT002_ALERT_2_G Number of Distance or Angle Restraints on AtSite          5 Note
PLAT003_ALERT_2_G Number of Uiso or Uij Restrained non-H Atoms ...         29 Report
PLAT007_ALERT_5_G Number of Unrefined Donor-H Atoms ..............          4 Report
PLAT042_ALERT_1_G Calc. and Reported MoietyFormula Strings  Differ     Please Check
PLAT072_ALERT_2_G SHELXL First  Parameter in WGHT  Unusually Large       0.33 Report
PLAT172_ALERT_4_G The CIF-Embedded .res File Contains DFIX Records          1 Report
PLAT178_ALERT_4_G The CIF-Embedded .res File Contains SIMU Records          2 Report
PLAT187_ALERT_4_G The CIF-Embedded .res File Contains RIGU Records          1 Report
PLAT188_ALERT_3_G A Non-default SIMU Restraint Value has been used     0.0050 Report
PLAT188_ALERT_3_G A Non-default SIMU Restraint Value has been used     0.0100 Report
PLAT190_ALERT_3_G A Non-default RIGU Restraint Value for First Par     0.0100 Report
PLAT190_ALERT_3_G A Non-default RIGU Restraint Value for SecondPar     0.0200 Report
PLAT300_ALERT_4_G Atom Site Occupancy of N13       Constrained at         0.5 Check
PLAT300_ALERT_4_G Atom Site Occupancy of N017      Constrained at         0.5 Check
PLAT300_ALERT_4_G Atom Site Occupancy of C1        Constrained at         0.5 Check
PLAT300_ALERT_4_G Atom Site Occupancy of C4        Constrained at         0.5 Check
PLAT300_ALERT_4_G Atom Site Occupancy of C9        Constrained at         0.5 Check
PLAT300_ALERT_4_G Atom Site Occupancy of C10       Constrained at         0.5 Check
PLAT300_ALERT_4_G Atom Site Occupancy of C017      Constrained at         0.5 Check
PLAT300_ALERT_4_G Atom Site Occupancy of C01J      Constrained at         0.5 Check
PLAT300_ALERT_4_G Atom Site Occupancy of C01F      Constrained at         0.5 Check
PLAT300_ALERT_4_G Atom Site Occupancy of C24       Constrained at         0.5 Check
PLAT300_ALERT_4_G Atom Site Occupancy of C01I      Constrained at         0.5 Check
PLAT300_ALERT_4_G Atom Site Occupancy of C01D      Constrained at         0.5 Check
PLAT300_ALERT_4_G Atom Site Occupancy of C01G      Constrained at         0.5 Check
PLAT300_ALERT_4_G Atom Site Occupancy of C018      Constrained at         0.5 Check
PLAT300_ALERT_4_G Atom Site Occupancy of H1        Constrained at         0.5 Check
PLAT300_ALERT_4_G Atom Site Occupancy of H22       Constrained at         0.5 Check
PLAT300_ALERT_4_G Atom Site Occupancy of H25       Constrained at         0.5 Check
PLAT300_ALERT_4_G Atom Site Occupancy of H01L      Constrained at         0.5 Check
```

```
PLAT300_ALERT_4_G Atom Site Occupancy of H26        Constrained at        0.5 Check
PLAT300_ALERT_4_G Atom Site Occupancy of H01M       Constrained at        0.5 Check
PLAT300_ALERT_4_G Atom Site Occupancy of H27        Constrained at        0.5 Check
PLAT300_ALERT_4_G Atom Site Occupancy of H01N       Constrained at        0.5 Check
PLAT300_ALERT_4_G Atom Site Occupancy of H01P       Constrained at        0.5 Check
PLAT300_ALERT_4_G Atom Site Occupancy of H018       Constrained at        0.5 Check
PLAT300_ALERT_4_G Atom Site Occupancy of H30        Constrained at        0.5 Check
PLAT300_ALERT_4_G Atom Site Occupancy of H31        Constrained at        0.5 Check
PLAT300_ALERT_4_G Atom Site Occupancy of O01E       Constrained at        0.7 Check
PLAT300_ALERT_4_G Atom Site Occupancy of H01Q       Constrained at        0.7 Check
PLAT300_ALERT_4_G Atom Site Occupancy of H01R       Constrained at        0.7 Check
PLAT302_ALERT_4_G Anion/Solvent/Minor-Residue Disorder (Resd  2  )       100% Note
PLAT302_ALERT_4_G Anion/Solvent/Minor-Residue Disorder (Resd  3  )       100% Note
PLAT302_ALERT_4_G Anion/Solvent/Minor-Residue Disorder (Resd  4  )       100% Note
PLAT302_ALERT_4_G Anion/Solvent/Minor-Residue Disorder (Resd  5  )       100% Note
PLAT302_ALERT_4_G Anion/Solvent/Minor-Residue Disorder (Resd  6  )       100% Note
PLAT304_ALERT_4_G Non-Integer Number of Atoms in ..... (Resd  3  )       2.09 Check
PLAT304_ALERT_4_G Non-Integer Number of Atoms in ..... (Resd  4  )       1.56 Check
PLAT304_ALERT_4_G Non-Integer Number of Atoms in ..... (Resd  5  )       1.35 Check
PLAT304_ALERT_4_G Non-Integer Number of Atoms in ..... (Resd  6  )       2.10 Check
PLAT432_ALERT_2_G Short Inter X...Y Contact  Cl2      ..C00S     .       3.24 Ang.
                                              -x,1-y,1-z  =      2_566 Check
PLAT432_ALERT_2_G Short Inter X...Y Contact  Cl2B     ..C00R     .       3.23 Ang.
                                              1+x,y,-1+z  =      1_654 Check
PLAT432_ALERT_2_G Short Inter X...Y Contact  Cl4B     ..C010     .       3.21 Ang.
                                                   x,y,z  =      1_555 Check
PLAT432_ALERT_2_G Short Inter X...Y Contact  O5       ..C24      .       2.96 Ang.
                                                 1+x,y,z  =      1_655 Check
PLAT432_ALERT_2_G Short Inter X...Y Contact  C00O     ..C10      .       3.15 Ang.
                                                   x,y,z  =      1_555 Check
PLAT605_ALERT_4_G Largest Solvent Accessible VOID in the Structure        114 A**3
PLAT720_ALERT_4_G Number of Unusual/Non-Standard Labels ..........         70 Note
PLAT789_ALERT_4_G Atoms with Negative _atom_site_disorder_group  #         26 Check
PLAT790_ALERT_4_G Centre of Gravity not Within Unit Cell: Resd.  #          2 Note
              C12 H12 N2
PLAT790_ALERT_4_G Centre of Gravity not Within Unit Cell: Resd.  #          6 Note
              H2 O
PLAT811_ALERT_5_G No ADDSYM Analysis: Too Many Excluded Atoms ....          ! Info
PLAT822_ALERT_4_G CIF-embedded .res Contains Negative PART Numbers          2 Check
PLAT860_ALERT_3_G Number of Least-Squares Restraints .............        229 Note
PLAT869_ALERT_4_G ALERTS Related to the Use of SQUEEZE  Suppressed          ! Info
PLAT933_ALERT_2_G Number of HKL-OMIT Records in Embedded .res File          7 Note
PLAT941_ALERT_3_G Average HKL Measurement Multiplicity ...........        2.8 Low
```

---

0 **ALERT level A** = Most likely a serious problem - resolve or explain
1 **ALERT level B** = A potentially serious problem, consider carefully
8 **ALERT level C** = Check. Ensure it is not caused by an omission or oversight
66 **ALERT level G** = General information/check it is not something unexpected

1 ALERT type 1 CIF construction/syntax error, inconsistent or missing data
15 ALERT type 2 Indicator that the structure model may be wrong or deficient
8 ALERT type 3 Indicator that the structure quality may be low
49 ALERT type 4 Improvement, methodology, query or suggestion
2 ALERT type 5 Informative message, check

It is advisable to attempt to resolve as many as possible of the alerts in all categories. Often the minor alerts point to easily fixed oversights, errors and omissions in your CIF or refinement strategy, so attention to these fine details can be worthwhile. In order to resolve some of the more serious problems it may be necessary to carry out additional measurements or structure refinements. However, the purpose of your study may justify the reported deviations and the more serious of these should normally be commented upon in the discussion or experimental section of a paper or in the "special_details" fields of the CIF. checkCIF was carefully designed to identify outliers and unusual parameters, but every test has its limitations and alerts that are not important in a particular case may appear. Conversely, the absence of alerts does not guarantee there are no aspects of the results needing attention. It is up to the individual to critically assess their own results and, if necessary, seek expert advice.

Publication of your CIF in IUCr journals

A basic structural check has been run on your CIF. These basic checks will be run on all CIFs submitted for publication in IUCr journals (*Acta Crystallographica*, *Journal of Applied Crystallography*, *Journal of Synchrotron Radiation*); however, if you intend to submit to *Acta Crystallographica Section C* or *E* or *IUCrData*, you should make sure that full publication checks are run on the final version of your CIF prior to submission.

Publication of your CIF in other journals

Please refer to the *Notes for Authors* of the relevant journal for any special instructions relating to CIF submission.

PLATON version of 10/05/2023; check.def file version of 10/05/2023

Datablock a_sq - ellipsoid plot

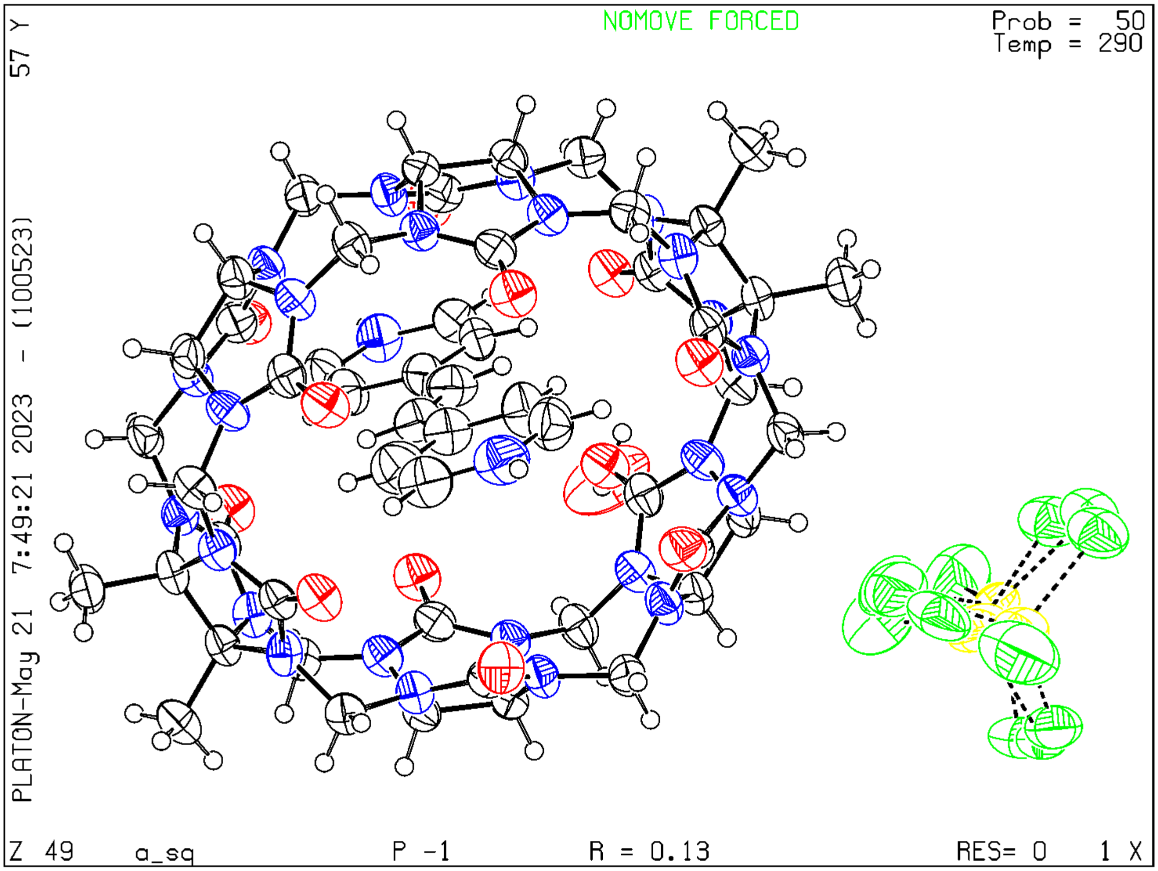

Supplement: File 3 — CheckCIF/PLATON report for the cif file of G@TMeQ[6]. [file Beilstein_J_Org_Chem-19-864-s003.pdf]
